# Revisiting the Trivers-Willard theory on birth sex ratio bias: Role of paternal condition in a Malagasy primate

**DOI:** 10.1371/journal.pone.0209640

**Published:** 2018-12-21

**Authors:** Martine Perret

**Affiliations:** UMR Mecadev 7179 CNRS-MNHN, Département Adaptations du Vivant, Brunoy, France; University of Missouri Columbia, UNITED STATES

## Abstract

Within current theories on potential adaptive manipulation of offspring sex ratio, giving birth to a male or to a female is assumed to depend on the capacity of the mother to invest in offspring to maximize her fitness. The active role of the father in sex ratio bias at birth has been neglected until recently. The human sex ratio at birth is biased towards sons, although in occidental populations, the ratio has decreased regularly for 30 years and could be the consequence of the adverse effects of environmental chemicals on male hormones. In a Malagasy primate, the lesser mouse lemur, the potential effect of paternal testosterone levels on sex ratio bias at birth was tested on 130 litters (278 babies) produced in 52 mixed-sex groups. For each group, social dominance among males was characterized based on aggressive interactions and sexual behaviours. Using a multi correspondence analysis, high testosterone levels in grouped males, particularly those of the dominant male, were significantly correlated with more infants produced in male-biased litters, independent of the female condition. According to these results, predictions for sex ratio bias towards one sex or the other in mouse lemurs were discussed considering the influence of both parents.

## Introduction

As a key for life history strategies, parental ability to control the sex of the offspring should provide an evolutionary advantage and therefore, has been studied in a variety of taxa. The Trivers-Willard theory (TW) [1] predicts that the sex ratio of offspring (SR, male births/total births) should vary with the capacity of the mother to invest in her offspring to maximize her lifetime reproductive success. Besides the TW theory, an alternative hypothesis proposes that competition for local resource (LRC) which acts on individuals within groups may shape birth sex ratios of individuals based on their condition [2,3]. More, the theoretical scenario developed by Leinar [4] suggests that high quality females should produce the sex whose reproductive success is mostly influenced by maternal investment, when taking into account the lifetime reproductive values of sons or daughters. Other models stressed the role of sex–specific demography in sex ratio bias [5–8]. Thus, theoretical or empirical studies suggest that, the sex ratio biases are the result of facultative response of individuals depending on environmental constraints. However, whatever the model, the relationship between maternal quality, generally assessed by the social dominance, and offspring sex ratio remains the studied cue, the influence of the father’s quality being unknown or considered as negligible. Thus, despite several analyses on birth sex ratios among primate’s populations, including humans [9–13], no consistent pattern emerges from predictions and observations, suggesting more complex mechanisms for potential adaptive manipulation of offspring sex ratios. Primarily based only on the maternal condition, in the absence of the exact role of paternal conditions, our understanding of the selective forces affecting birth sex ratio is very restricted. However, high paternal concentrations of testosterone around the time of conception are suspected to skew offspring sex ratios towards sons [14, 15]. Understanding of the paternal role in sex ratio bias has come from indirect evidence. In the red deer, a positive correlation has been found between male fertility assessed by the percentage of normal spermatozoa and the production of sons [16]. In Human, the sex ratio at birth, normally biased towards males, has been in constant decline in industrialized countries over recent decades [17, 18]. Simultaneously, explorations on causes for the decline have stressed the widespread decrease in semen quality (motility, concentration, ratio of Y:X spermatozoids) in industrialized countries [19, 20], suggesting that the significant overall decline in male births could be linked to a negative effect of environmental factors on male reproductive function. Supporting this hypothesis, stress from multiple origins is correlated with a low sex ratio, including an earthquake [21], the Slovenian war [22], industrial pollution [23], socioeconomic conditions [24–27], and terrorist attacks [28]. Recently, environmental endocrine-disrupting chemicals, for example, perfluoroalkyl substances (PFAS), bisphenol (BPA) and pesticides, have been shown to affect parental hormones and the secondary sex ratio through changes in sperm morphology and motility [29–31]. When both parents are exposed to PFAS, BPA or other chemical substances, an excess of female births is observed that is significantly linked to only paternal chemical concentrations [32–34]. Thus, endocrine-disrupting chemicals have clear anti-androgenic activities, as demonstrated in most animal studies [35–37] and in adult humans [38–40]. However, to determine whether the alterations of human sperm are correlated with low fertility and low sex ratio at birth, a direct relationship remains to be established between sperm quality and testosterone levels and a decrease in birth sex ratio [31, 34, 41].

The low fecundity of most primates is one difficulty in elucidating the role of the father condition in sex ratio bias. By contrast, the reproductive patterns and high fecundity of the grey mouse lemur, a small Malagasy primate (*Microcebus murinus*), offer several practical advantages for experimentally testing the bias of sex ratio at birth. Reproduction is highly seasonal with females exhibiting marked oestrous synchrony at the beginning of the reproductive season. Females give birth to 1–3 young per litter, exceptionally 4, after a gestation period lasting approximately 2 months. With regards to males, field studies and captive observations revealed that the mating system is mainly based on polygynous-polyandrous strategies with male guarding behaviour and female choice [42–45]. A male-biased sex ratio at birth is observed in captive mouse lemur females (51.3% from more than 2300 births). Skewed sex ratios were previously demonstrated to occur at a conception level, with the ratios independent of the female nutritional state or litter size [46]; however, birth sex ratios are dependent on social interactions among females during the follicular phase [47] through changes in oestrogen levels before oestrus [48]. When maintained in large uni-sex groups before mating, females overproduced sons, whereas isolated females presented the inverse tendency. When kept in a heterosexual group before mating, the sex ratio at birth remains slightly male-biased.

However, these results were obtained without considering the potential role of the father in the direction of the sex ratio bias. Using genetic paternity analysis in wild animals, heavy males with high competitive abilities sire more offspring [42, 43]. In captivity, a high sexual competition for prior access to oestrous females develops among males, leading to the emergence of a dominant male who fathers almost all the offspring produced [49, 50]. Thus, the aim of this study was to examine the relationship between characteristics of males immediately before mating within a group (dominance, body mass, age and testosterone level) and litter production in terms of number and sex of newborns. Then, a model based on both male and female hormonal conditions was proposed for the direction of sex ratio at birth in this primate.

## Materials and methods

### Animals

The experiments covered a period of seven years, and all mouse lemurs used in this study (156 males and 156 females) were born in the breeding colony established at Brunoy (France, IBISA platform, agreement E91.114.1, DDPP Essonne) from a stock originally caught near the southern coast of Madagascar fifty years ago. Captive conditions were maintained constant with respect to ambient temperature (24–26°C) and hygrometry (55–60%). Animals were fed *ad libitum* on a standardized diet, including fresh fruits, a homemade mixture and mealworms. To ensure seasonal reproductive rhythms, animals were routinely exposed to an artificial photoperiodic cycle consisting of 6 months of summer-like photoperiod (14 h of light/day) followed by 6 months of winter-like photoperiod (10 h of light/day). The beginning of the breeding season was induced by the exposure to long days [51].

### Experimental design

Except during the experiments, males and females were kept in single sex groups of 2–3 animals per cage. At the beginning of the breeding season induced by the long days, groups of 3 males and 3 females each were constituted and kept in cages (180x150x120 cm) with many wooden supports and several nest boxes. Both males and females were weighed before the observation period and were chosen so that individuals within a group were not closely related (first-order relatedness such as parentage of full sib ship). Within a group, males and females were of a similar body mass (average difference 9.5 ± 3 g) and age (average difference 0.2 ± 0.1 year). All oestrus occurred synchronously within a 10-day period, and individual behaviours (agonistic and sexual interaction) within each group were video recorded using an infrared camera (MediaZoomIR1; AXOS, France) during the first 6 hours of nocturnal activity [49, 44, 52]. Genetic determinations of paternity demonstrate a highly robust relationship between the rank of a male and its reproductive success [49]. Consequently, a male dominance index was calculated based on the outcomes of aggressive interactions won (w) or lost (l) during the total observation period using the formula (w-l) / (w+l). In each group, males were ranked according to the decreasing value of the dominance index from +1 for a male whose agonistic interactions were always successful (α) to -1 for a male that never initiated fights and was always chased by the two other males (γ). This dominance index [49, 50] that assesses a rank for each male in a group allows the comparison of animals living in different social groups. For females, no clear social rank could be detected because of the very low agonistic interactions between them during the short mating period.

For males, immediately before the observation period, a blood sample (100 μl) was drawn via the saphenous vein without anaesthesia 4 h before night. The sample was immediately centrifuged and stored at –20°C until assayed. Plasmatic testosterone was measured in duplicate using an ELISA immunoenzymoassay (RE52041; IBL, Hamburg, Germany), using a method previously described [53]. The mean intra-assay coefficient of variation was 4.9%, and the mean inter-assay coefficient of variation was 3.7%.

After the occurrence of oestrus, females were withdrawn from the group, isolated and checked for gestation 3 weeks later. At birth, size and sex ratio of the litter were recorded. According to the sex of each newborn produced (M = male, F = female; visually distinct from birth), the type of the litter was defined as well balanced for MF, MMFF litters, male-biased for M, MM, MMF and MMM litters and female-biased for F, FF, FFM and FFF litters.

### Statistical analyses

Data are presented as the mean ± SEM. For each group, means of body mass and age for both males and females were calculated. Additionally, mean testosterone levels per group, mean female parity and the total number of offspring produced by group were analysed. Statistical analyses included multi-way analysis of variance or analysis of covariance with body mass or age. Non-parametric tests for mean difference according to the normality of the data and linear correlations between parameters were also used. Between-group variation with respect to age, body mass in both males and females, testosterone value, dominance rank, and number and sex of offspring produced was analysed using a centred-scaled principal component analysis (N = 52 groups) computed in R using the ‘FactorminR’ 1.28 package. For statistical analysis of sex ratios, we compared the proportions of litters in which infants in one sex predominated (male-biased or female-biased), irrespective of the litter size, with those of well-balanced litters.

### Ethics statement

We adhered to the Guidelines for the Treatment of Animals in Behavioural Research and Teaching [54] and the legal requirements of the country (France) in which the work was conducted. All the results in this study did not correspond to experimental procedures but are issued from the exploitation of the reproductive data collected in the long-term mouse lemur’s captive population maintained for scientific purposes. This colony is established under the authorisation of the *Direction Départementale de Protection des Populations* (DDPP/022-E91-114-1) and is affiliated to the Ethical Comity Cuvier (C2EA 68). All procedures to breed mouse lemurs are conducted in accordance with the European Communities Council Directive (86/609/EEC) and are performed by authorized animal breeders delivered by the Departmental Veterinary Services (Directive 2010/63/UE–Capacity certificate *Préfecture de l’Essonne*, 04/03/1995). Specifically for reproduction, housing conditions enable the animals to express their entire locomotion and sexual repertoire. Several nest-boxes are provided so that animals could escape agonistic interactions from conspecifics, and a chase or a fight immediately stops when the chased animal enters a nest-box. None of the animals used in this study was injured, and in the colony, heterosexual groups were restricted in time to the few days of female oestrus. Lastly, no anaesthesia, euthanasia or animal sacrifice is part of this study.

## Results

### Reproductive parameters of males

**Fig 1:**
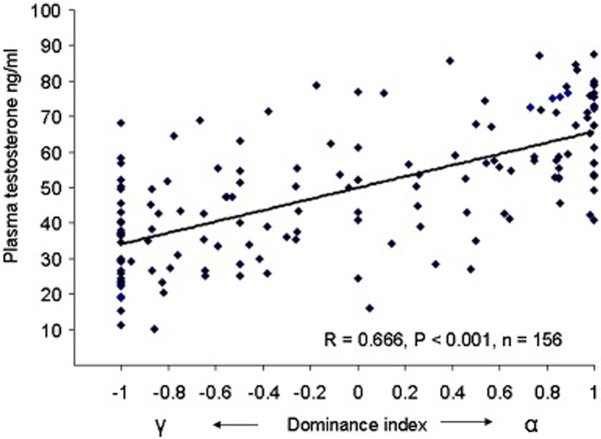
Relationship between plasma testosterone levels and dominance index based on aggressive interactions between males within each group.

Body mass and age of the 156 studied males were on average 90 ± 2 g and 2.5 ± 0.1 years, respectively. For all males, significant correlations were observed between body mass, testosterone levels, age and the dominance index (Table 1). Dominance status were positively linked to testosterone levels (F_1/151_ = 48.4, P < 0.001 –Fig 1), but not to age (P = 0.36) nor to body mass (P = 0.08) without interactions between variables (P = 0.35). Within each group, independently of body mass and age, dominant males exhibited higher testosterone levels (64.5 ± 1.8 ng/ml) compared to subordinate males (42.4 ± 1.6 ng/ml, *F*_1/154_ = 71.6, P < 0.001).

**Table 1:** Characteristics of the 156 studied males with correlation between parameters.

	μ ± sem	Body mass	Testosterone	Age
Body mass (g)	90 ± 2.4			
Testosterone	49.5 ± 1.3	0.2437 **P = 0.002**		
Age (years)	2.44 ± 0.1	0.1338 P = 0.08	0.0742 P = 0.36	
Dominance Index	-0.02 ± 0.06	0.2565 **P = 0.001**	0.6667 **P < 0.001**	0.1682 **P = 0.036**

### Reproductive parameters of females

Body mass and age of the 156 studied females were on average 99 ± 1.5 g and 2.7 ± 0.1 years, with no significant correlation between age and body mass (r = 0.0842, P = 0.29). Fifty-three females were primiparous, whereas the others had 1 to 4 successful pregnancies before the experiment. Within the grouped females, 26 females did not enter pregnancy, mainly multiparous females (23%). A multivariate analysis demonstrated that the female reproductive success was not influenced by body mass (P = 0.76), by age (P = 0.15) or by parity (P = 0.14) with no interactions between these parameters (P = 0.14).

One hundred thirty litters were produced with a general male-biased sex ratio at birth of 56.5% (157 males/121 females), significantly different from 50% (X^2^ = 4.66, P < 0.05). On average, females gave birth to 2.1 ± 0.05 babies per litter (range 1 to 3, exceptionally one litter of 4). The size of the litters followed the usual pattern observed for mouse lemurs, i.e., with almost 50% of litters with 2 babies (69/130), and this distribution did not differ according to parity (*F*_1/126_ = 0.04, P = 0.85), body mass (*F*_1/126_ = 0.94, P = 0.15) or age (*F*_1/126_ = 2.09, P = 0.40), although the primiparous females were significantly younger than the multiparous females (*F*_1/128_ = 58.3, P < 0.001). The type of the litters (F, FF, FFF, MF, M, MM, MMM, MMF, MFF, MMFF) was statistically independent of body mass (*F*_9/120_ = 0.834, P = 0.58), parity (*F*_9/120_ = 0.52, P = 0.79) or age (*F*_9/120_ = 089, P = 0.52).

When considering the bias in litters, male-biased, female-biased or well balanced, a deficit in female-biased litters was observed (X_2_ = 28.7, df 1, P < 0.001) that was independent of female parity (*F*_2/127_ = 1.01, P = 0.36), body mass (*F*_2/127_ = 0.42, P = 0.665) or age (*F*_2/127_ = 1.36, P = 0.26). The general male-biased sex ratio within females appeared primarily because of a significant deficit of litters with 2 female babies (X^2^ = 28.7 df 2, P < 0.001), regardless of female parity, body mass or age. Thus, the number and the sex of the infants produced per female did not significantly correlate with body condition (Table 2).

**Table 2:** Characteristics of the studied females (means ± SEM). Significant differences between primiparous and multiparous females are indicated.

	Non pregnant	Primiparous	Multiparous
N	26	55	75
Body mass (g)	95 ± 3	97 ± 3	102 ± 2 p = 0.11
Age (year)	2.9 ± 0.2	1.7 ± 0.1	3.3 ± 0.1 **P < 0.01**
Parity	2.2 ± 0.1	1	2.7 ± 0.07 P = 0.95
Nb babies / litter		2.13 ± 0.1	2.12 ± 0.07 P = 0.95
Sex ratio (% males)		53.0	59.0
Production		62 males / 55 females	95 males / 66 females

### Characteristics of sex-mixed groups and offspring sex ratio

Before testing the possible relationship between testosterone levels in males and sex ratio bias at birth, the homogeneity of the different parameters (body mass, age, reproductive success) within the 52 groups was tested according to the type of the offspring sex ratio produced by each group. The infants produced by each of the 52 groups were distributed as well balanced (N = 8, 18M-18F), female-biased (N = 16, 22M–61F) and male-biased (N = 28, 115M-44F). This distribution differed from that of the theoretical one (X_2_ = 4.06, P < 0.01), corresponding to a clear deficit in female-biased production. Because of the choice of sexual partners in the experimental design, no significant difference was observed for any parameter representative of body condition for both males and females (Table 3). However, male-biased sex ratio was correlated with testosterone levels recorded in the dominant male (*F*_2/49_ = 14.3, P < 0.001; r = 0.6342, N = 52, P < 0.001), but not with mean of testosterone levels in subordinate males (*F*_2/49_ = 0.83, P = 0.439; r = 0.211, N = 52, P = 0.13). Lastly, no significant relationship was observed between testosterone levels of males and the number of non-successful females within a group (P = 0.10).

**Table 3:** Mean ± SEM per group of sexual partners’ characteristics according to the type of litters:–male biased (M),—well balanced (E) and,–female-biased (F). The only one significant difference was underlined in bold.

Mean ± SEM per group	M	E	F	P value
N	28	8	16	
SexRatio %	**76.3 ± 3**	**50**	**21.7 ± 3.9**	** **
Body mass Males(g)	91 ± 3	99 ± 8.3	84 ± 4.5	P = 0.11
Body mass Females (g)	98 ± 3	103 ± 6	100 ± 4	P = 0.61
Males age (year)	2.3 ± 0.2	2.7 ± 0.3	2.6 ± 0.2	P = 0.30
Females age (year)	2.6 ± 0.2	2.8 ± 0.4	2.7 ± 0.2	P = 0.73
% success mating	90.5 ± 4.2	85.5 ± 8	74 ± 7	P = 0.09
Parity	1.95 ± 0.1	2.4 ± 0.2	2.06 ± 0.1	P = 0.09
Total offspring produced	5.7 ± 0.4	4.5 ± 0.7	5.3 ± 0.8	P = 0.49
Number of young / Female	2.1 ± 0.08	2.0	2.3 ± 0.2	P = 0.42
Testosterone subordinate males(ng/ml)	44.9 ± 2.3	44.5 ± 4.2	43.6 ± 1.9	P = 0.44
Body mass dominant males (g)	96 ± 4	107 ± 10	87 ± 6	P = 0.148
Age dominant males (year)	2.5 ± 0.2	2.8 ± 0.4	2.9 ± 0.3	P = 0.46
Testosterone dominant males (ng/ml)	**71.3 ± 2**	**60.2 ± 4.2**	**53.3 ± 2.7**	**P < 0.001**

**Fig 2:**
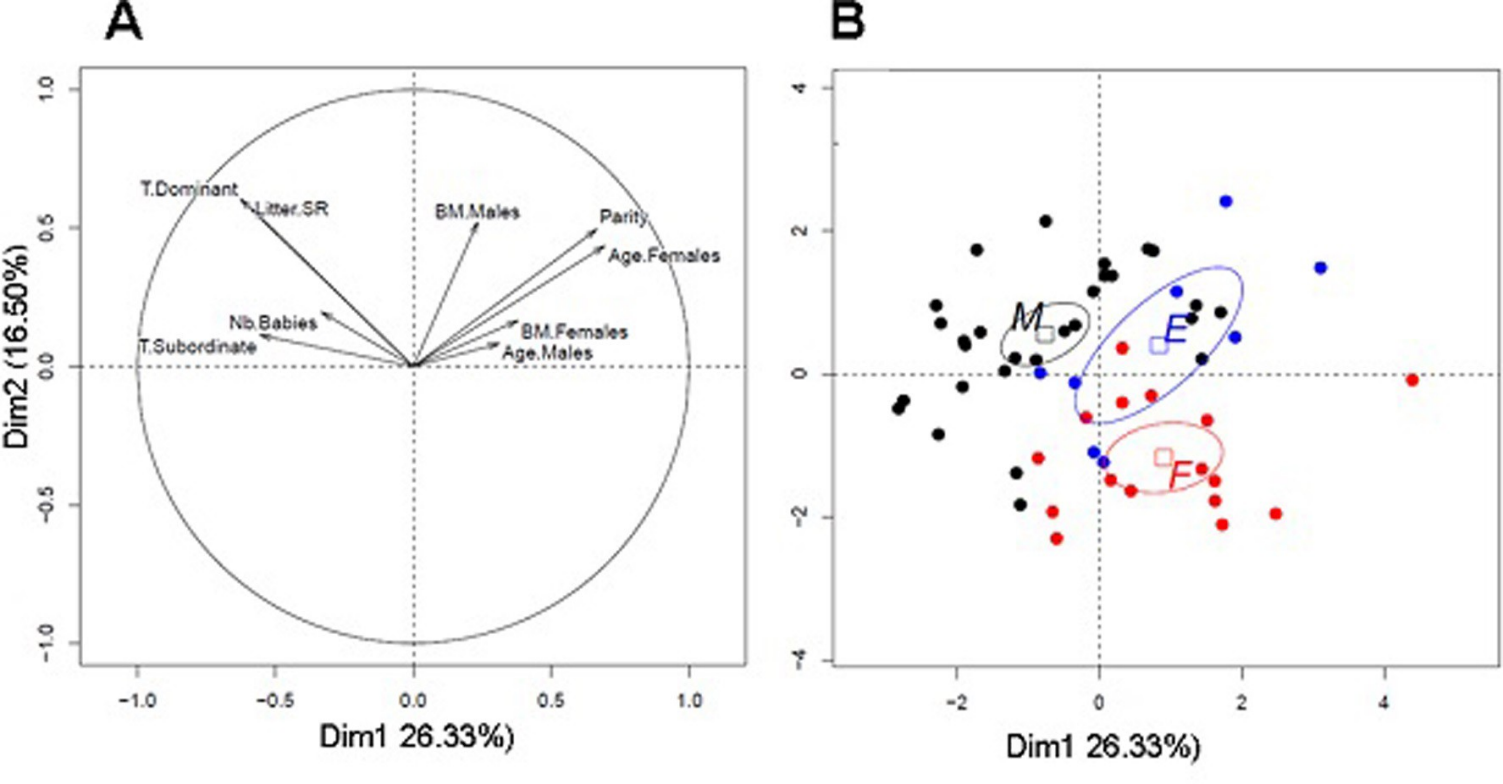
Principal component analysis. **(A)—**Variable factor map on the 52 tested groups with quantitative variables:—body mass (BM) and age of both males and females,—parity,—testosterone levels (T) for the dominant male and the subordinate males in each group, -litter sex ratio and number of babies produced. Variables are represented by their scores on Dim1 (x-axis) and Dim2 (y-axis). The X and Y axis represent principal component loadings on Dim1 and Dim2 respectively, which corresponded to the weights of each original variable graphically visualized by arrows. Dim1 (26.3%) represents the influence of parents ‘ body condition on the litters data among which the parity and age of the grouped females were the primary affecting factor. Dim2 (16.5%) shows the strong relationship between testosterone levels of the dominant male and offspring production biased towards males. (B)—Group factor map on the 52 tested groups with litter types as qualitative variable. PCA clearly separates groups producing male-biased litters (M, black) from groups producing female-biased litters (F, red circles). Well-balanced litters appeared intermediate (E, blue).

Principal component analysis was performed on the 52 groups with the type of the litter (E, F and M) as the qualitative variable and as quantitative variables: body mass and age of sexual partners, female parity, testosterone level of dominant male, testosterone level of subordinate males, the sex ratio and the total number offspring produced per group. The first two principal components of the variable map (Fig 2A) explained 43% of the total variation in birth sex ratio determination. The first axis (Dim 1, 26.3%) mainly received positive loadings (near 70%) from female age and parity (linked together). The second axis (Dim 2) accounted for 16.5% and primarily received positive and similar loadings (near 60%) for litter sex ratio and testosterone levels of dominant males. Both axes received weak and comparable loadings (less than 20%) from body mass of females, age of males, testosterone levels of subordinate males and the number of babies produced. Finally, PCA confirmed that male-biased litters are primarily linked to testosterone levels of dominant males, independently of parents’ body condition or age.

The group factor map (Fig 2B) showed the repartition of groups according to the litter type they produced. The Dim 1 axis received positive loadings (60%) from groups producing female-biased litters, clearly opposed to groups producing male-biased litters. Well-balanced litters remained intermediate. The discrimination might have been better with more numerous well-balanced litters; however, a PCA without integrating the well-balanced litters produced the same results.

Lastly, a slight but significant correlation (r = 0.2795, P = 0.044) was detected between mean testosterone levels in a group and the number of babies produced. When mean testosterone levels of males in a given group were high, the sexual competition seemed stronger accounting for more numerous infants born in male-biased litters.

## Discussion

Mouse lemurs are a promiscuous and monomorphic primate species. In the wild, females share their home range with their daughters, whereas males disperse [55]. According to the Local Resource Competition theory of sex allocation, an increase in competition between philopatric females for local resources and mates should favour a skewed sex ratio at birth towards males [2, 4, 56]. Experimental studies demonstrate that the social context in which females live before mating plays a major role in determining offspring sex ratio. Isolated females produce more daughters; unisex-grouped females produce more sons; whereas sex-mixed grouped females gave birth to a near equilibrated sex ratio, with these differences occurring at a conception level without sex-selective abortions [46]. Differences have been linked to changes in hormonal conditions of females before conception, mediated by urinary olfactory cues [47, 48]. In this study, to avoid differences in female status, the role of male condition was tested with females kept in the same social conditions, i.e., brief sex-mixed groups. Such conditions are known to limit potential social stress effects on female reproductive capacity [46]. Moreover, analyses revealed that the success of pregnancy and the number of babies born per female were independent of body condition or parity of females in all groups. This result provided confidence that differences observed in sex ratio biases among groups were primarily dependent on the male condition.

With regards to mouse lemur’s males, field studies on mating strategies have long suggested the importance of scramble competition among males and pronounced sperm competition [42, 43, 57]. Later, direct observations of mating behaviour revealed the neglected role of male guarding behaviours and that of female choice [43–45]. In captivity, the potential for a male to monopolize mating is relatively high and sexual competition through fights and pheromonal inhibition of rivals leads to a clear hierarchy within grouped males [50, 58]. Usually only the dominant males mate females, because females preferentially accept copulations from dominant and aggressive males [44, 45, 49]. Although all babies born in this study cannot be assumed unique descendants from the dominant male in each group, previous genetic paternity assessments reveal that 93% of babies issued from a group were fathered by the dominant male (50, 58]. In the wild, local competition for oestrous females leads to a transient hierarchy among males [59], and the dominant male obtains priority access to females ensuring his reproductive fitness will increase, as observed in many gregarious primates (review [60]). Based on the relationship in this study between mean testosterone level of males in a group (particularly that of the only dominant male) and the sex ratio of newborns produced, an effect of male hormonal condition was strongly suggested. This hypothesis is consistent with previous studies suggesting that siring a male offspring is linked to father reproductive quality estimated by sperm quality [16] or in fertility treatments known to increase testosterone levels [13]. Lastly, high testosterone levels of males within a group led to a significant increase in the number of newborns produced, potentially linked to the stimulating effects of testosterone-dependent pheromones on ovulation’ rate in mouse lemur females.

**Fig 3:**
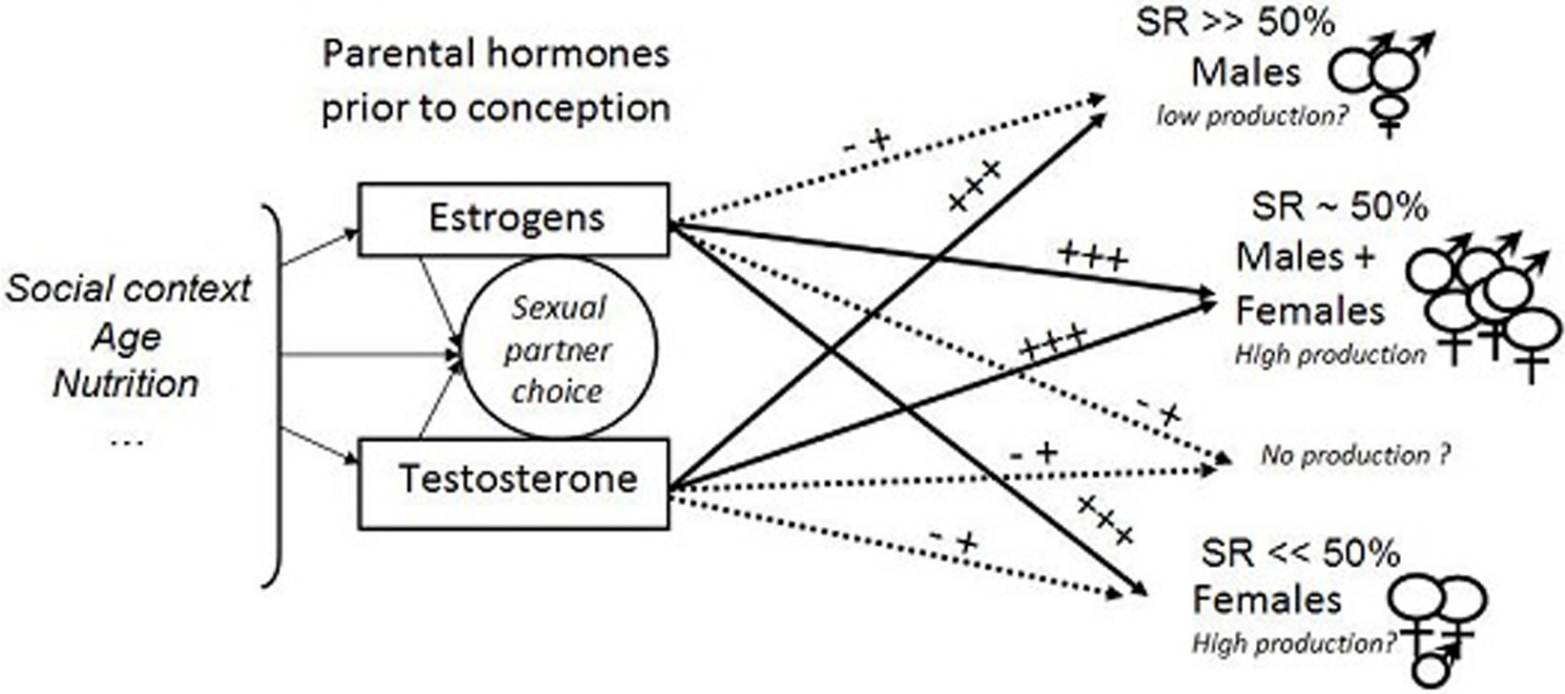
–Predictions on the direction of sex ratio (SR) bias et potential production in mouse lemurs. According to the level of parents’ sexual hormones prior to conception (+++ good or +- low), the future reproductive fitness of offspring ♀ and ♂ might be low (small symbol) or good (large symbol) depending on the sex ratio of the litter.

In several studies, whether the variations in sex ratio bias between species or within species were consistent with current theories likely depended on environmental conditions, particularly resource availability. In captive mouse lemurs, access to food is not limited, and therefore, the skewed sex ratios observed for one or the other sex primarily depended on parental interactions. Considering that maternal and paternal hormonal conditions might have additive effects in determining offspring sex ratio, a schematic model was constructed to predict the direction of the bias in sex ratio at birth in this primate (Fig 3). Because body mass of parents was apparently not a predicting factor for the skewed sex ratios ([46], and this study), we focused on sexual hormones.

Depending both on the environmental context (resources, population density, social interactions) and intrinsic factors (age, health), hormonal conditions of parents before conception may be high or low, and as a first consequence, success of mating behaviour will be potentially affected. Indeed, females prefer males with high aggressive potential (i.e., high testosterone levels); whereas males prefer females with high oestrogen levels [44]. Then, reproductive success will be high when both parents are in good hormonal condition and most likely low, even absent, when parents are in poor hormonal condition. Females with high oestrogen levels will be highly attractive and will choose the most competitive male (local dominance) among the males in competition. These females will produce numerous babies, and the offspring sex ratio will be skewed to one sex or the other depending on the male hormonal condition: bias towards males with a good father or towards females when mated with a less suitable father. By contrast, females with low oestrogen levels will be less attractive and when mated, will give birth to few offspring with a male-biased sex ratio. Such model taking into account father quality, even without paternal care, may explain the bias of sex ratio towards one sex or another for high quality females, independently of the context. Lastly, the litter type can affect the growth and survival of infants, suggesting a potential effect on the future reproductive fitness of offspring produced (Fig 3).

However, limiting explanations for offspring sex ratio bias to only hormonal status of both parents could lead to misinterpretation. In various species, the condition of parents has been assessed based on only the dominance rank or on some morphological/physiological characteristic, although many other factors are involved. Nevertheless, because the condition of parents around conception has unanimous support for determining a conceptual sex bias, studies are focused on the female physiological state around conception. In addition to the effects of nutrition, other factors have been examined for their influence, such as maternal androgens or glucose levels before conception [13, 61–66].

Lastly, one of the primary questions from an evolutionary perspective concerns the potential fitness benefit for parents to produce one sex or the other [5, 6, 60]. Because paternal care does not occur in mouse lemurs, the reproductive value of the offspring is mostly influenced by mother quality and environment during early life. For example, litter sex composition in marmots affects life history traits and future dominance status [67, 68]. Preliminary results with captive mouse lemurs show that the reproductive value of male or female offspring varies according to the litter composition (unpublished data). High yearly turnover rates for both sexes are observed in wild populations of mouse lemurs with only 7% of females and 14% of males remaining alive at the onset of the third breeding season [69]. As a consequence, manipulation of sex ratio at birth and maternal investment might have important effects on the fitness benefit of parents in this species. Therefore, to assess the adaptive manipulation of offspring sex ratios by parents to maximize their fitness, further studies should be conducted on sex ratio bias in which the reproductive value is assessed during the lifetime of offspring.

## Supporting information

S1 File The data set used in this study is available in the file “data proceedings–MS17-34926R2. (XLS)
